# Willingness to Vaccinate Against COVID-19: The Role of Health Locus of Control and Conspiracy Theories

**DOI:** 10.3389/fpsyg.2021.717960

**Published:** 2021-10-22

**Authors:** Vojtech Pisl, Jan Volavka, Edita Chvojkova, Katerina Cechova, Gabriela Kavalirova, Jan Vevera

**Affiliations:** ^1^Department of Psychiatry, Faculty of Medicine, University Hospital in Pilsen, Charles University, Pilsen, Czechia; ^2^Department of Psychology, Faculty of Social Studies, Masaryk University, Brno, Czechia; ^3^Memory Clinic, Department of Neurology, Second Faculty of Medicine, Motol University Hospital, Charles University, Prague, Czechia; ^4^International Clinical Research Center, St. Anne’s University Hospital Brno, Brno, Czechia; ^5^Center of Physical Education and Sport, Faculty of Education, University of West Bohemia, Pilsen, Czechia; ^6^Institute for Postgraduate Medical Education, Prague, Czechia

**Keywords:** COVID-19, pandemics, vaccination, willingness to get vaccinated, conspiracy theories, health locus of control, conspiracy mentality

## Abstract

Understanding the predictors of the willingness to get vaccinated against COVID-19 may aid in the resolution of current and future pandemics. We investigate how the readiness to believe conspiracy theories and the three dimensions of health locus of control (HLOC) affect the attitude toward vaccination. A cross-sectional study was conducted based on the data from an online survey of a sample of Czech university students (*n* = 866) collected in January 2021, using the multivariate linear regression models and moderation analysis. The results found that 60% of Czech students wanted to get vaccinated against COVID-19. In addition, 40% of the variance of willingness to get vaccinated was explained by the belief in the COVID-19-related conspiracy theories and the powerful others dimension of HLOC. One-sixth of the variance of the willingness to get vaccinated was explained by HLOC, cognitive reflection, and digital health literacy [eHealth Literacy Scale (EHEALS)]. HLOC and conspiracy mentality (CM) and its predictors are valid predictors of a hesitancy to get vaccinated against COVID-19. The campaigns promoting vaccination should target the groups specifically vulnerable to the conspiracy theories and lacking HLOC related to powerful others.

## Introduction

The vaccination campaign against COVID-19 was launched in December 2020 in the Czech Republic, with only half of the population willing to get vaccinated a month later (National Pandemic Alarm, 2021). Vaccination plays a major role in stopping the pandemics, while the cognitive, emotional, and social processes shape public compliance with protective measures, such as vaccination. The WHO (2020) highlights the importance of addressing the “infodemic” as a part of the pandemic response and scientists point to the importance of taking into consideration the social and behavioral factors (Van Bavel et al., 2020) and research that can “inform contextualized campaigns and information-sharing that will ultimately result in increased confidence in and uptake of available vaccines” (Machingaidze and Wiysonge, 2021, p. 1339). Such research needs to investigate how individuals gather and interpret information about and the reason for or against the vaccines, as the primary motivation to get vaccinated is related to the perceived costs and benefits for personal well-being (Solís Arce et al., 2021). Further, studying the predictors of vaccination intentions is important for understanding the reasons and beliefs behind vaccine refusal rather than blaming those who refuse them (refer to Williams, 2021). The intention to get vaccinated against COVID-19 (VAC) is, among other predictors, associated with the beliefs in the COVID-19-related conspiracy theories and about how human health is determined by *health locus of control* (HLOC), which are examined by the present study.

Health locus of control consists of three relatively independent dimensions: *internal* (the belief that health is determined by the internal factors and personal effort) and two external ones: the *powerful others dimension* summarizing the belief that health is determined by other persons, especially the medical personnel and family members, and last, *the chance dimension*, or the belief that health depends on chance, God, or destiny (Wallston et al., 1978). *Internal* dimension tends to be positively related to the health behavior, medication adherence, and self-reported health status and *chance* dimension to psychological distress and lack of adherence (Wallston, 2004; Grotz et al., 2011; Náfrádi et al., 2017; West et al., 2018). The role of the *powerful others* dimension is more complex, as it places health control in the hands of medical professionals and other people may yield different outcomes (Grotz et al., 2011; Náfrádi et al., 2017; West et al., 2018). Interaction of two dimensions or interaction of an HLOC dimension with another construct may play a crucial role (Wallston, 2004; O’Hea et al., 2005). With respect to vaccination, the *chance* dimension correlated with the vaccination intentions negatively (Chapman and Coups, 1999) and *powerful others* (Zhang et al., 2012; Kan et al., 2018) and *internal* HLOC (HLOC_I) (Tinsley and Holtgrave, 1989; Chapman and Coups, 1999) positively, although Kan et al. (2018) found opposite associations for the *chance* and *internal* HLOC, and the associations were not confirmed by Nexøe et al. (1999). In a recent model of the attitudes of parents toward child vaccination, the *internal* and *powerful others* HLOC is linked with pro-vaccination and *chance* HLOC is linked with the anti-vaccination attitudes (Aharon et al., 2018). Recently, the negative link between the *chance* of HLOC and the willingness to get vaccinated against COVID-19 was confirmed (Olagoke et al., 2021).

The intentions to vaccinate may be negatively affected by the conspiracy theories (Jolley and Douglas, 2014). In the case of COVID-19, a lack of willingness to get vaccinated was associated with the COVID-19-related conspiracy beliefs (Romer and Jamieson, 2020) and a gradual decrease in the vaccination intentions throughout 2020 was linked with the COVID-19-related misinformation (Robinson et al., 2021). Conspiracy theories are “attempts to explain the ultimate causes of significant social and political events and circumstances with claims of secret plots by two or more powerful actors” (Douglas et al., 2019, p. 4). The conspiracy claims, such as that COVID-19 is a hoax, or that it was spread intentionally, reduce compliance with protective measures and restrictions (Bierwiaczonek et al., 2020; Imhoff and Lamberty, 2020; Pummerer et al., 2021; as shown in Douglas, 2021 for an overview). Conspiracy mentality (CM) is studied as the individual predisposition to believe in conspiracy theories because beliefs in conspiracy theories from various domains are intercorrelated, even if the beliefs contradict each other (Wood et al., 2012; Imhoff and Bruder, 2013). CM is related to the external locus of control (Abalakina-Paap et al., 1999) and the belief in COVID-19-related conspiracy theories (Imhoff and Lamberty, 2020), and in those who perceive low support for the vaccination in their social environment, it predicts low vaccination intentions (Winter et al., 2021). CM is itself predicted by *dissociation* (Charlton, 2014) and *cognitive reflection*—the ability to reflect upon whether the result of an intuitive cognitive process is correct (Stoica and Umbreş, 2020). The COVID-19-related conspiracy theories are also associated with low *digital health literacy* (EHEALS) (Naeem and Boulos, 2021; Pickles et al., 2021), which is “the ability to seek, find, understand, and appraise health information from electronic sources and apply the knowledge gained to addressing or solving a health problem” (WHO, 2013, p. 61).

In our previous study on the same sample, we have shown that the COVID-19-related conspiracy theories were indeed predicted by digital health literacy, dissociation tendencies, and cognitive reflection and that the effect of the latter two was mediated by CM (Pisl et al., 2021). The present study further extends these results with respect to the vaccination intentions, studying the effects of HLOC and conspiracy theories and their predictors on the willingness to get vaccinated against COVID-19. Based on the model of Aharon et al. (2018), we hypothesize that the *internal* and *powerful others* HLOC is linked with higher and *chance* HLOC with the lower willingness of the university students of Czech to get vaccinated against COVID-19. With respect to the conspiracy theories, we examine how these conspiracy theories and their predictors influence the willingness to get vaccinated against COVID-19 in three steps. In the first model, we will test the effects of HLOC and belief in the COVID-19-related conspiracy theories on VAC. In the second model, we will test the effects of HLOC and the predictors of COVID-19-related conspiracy theories on VAC, expecting VAC to be related to the low CM and high digital health literacy. Further, we expect the effect of *internal* HLOC to interact with the CM and digital health literacy, indicating that the positive effect of *internal* HLOC on vaccination is higher in those who are well-informed and less susceptible to the conspiracy theories. In the third model, we will test the effects of HLOC, digital health literacy, and predictors of CM on VAC, expecting VAC to be related to low dissociation and high cognitive reflection.

## Materials and Methods

### Materials

**Health locus of control** was measured by the Multidimensional HLOC scale (MHLOC), version A (Wallston et al., 1978), a short instrument with acceptable reliability (Cronbach’s alpha usually hovers in the range 0.65–0.70) (Wallston, 2004), consisting of 18 items measuring three separate and only slightly intercorrelated dimensions: the belief that it is the subject who has control over their health (*internal* HLOC, HLOC_I), the belief that health of an individual is controlled by others (e.g., health professionals and family; *powerful others* HLOC, HLOC_P), and the belief that health is controlled by *chance* (HLOC_C). The answers were recorded on the 6-point Likert scales that were later converted to numbers ranging from 1 to 6 (6 meaning highest agreement), yielding three summary scores ranging from 6 to 36.

**The vaccination intention (VAC)** was measured by a single question: “How likely is it that you are going to get vaccinated against COVID-19” with 11 options ranging from 0 to 100%.

The measures of other variables were described previously by Pisl et al. (2021). **Experience with dissociation** was measured by the Dissociative Experience Scale (DES) (Ptáček and Bob, 2009), **CM** by the CM Questionnaire (CMQ) (Bruder et al., 2013), **cognitive reflection** by the cognitive reflection test (CRT) (Frederick, 2005), and digital health literacy (EHEALS) by the eHealth Literacy Scale (eHEALS) (Norman and Skinner, 2006). **The belief in two COVID-19-related conspiracy theories**, namely, that COVID-19 is a hoax, and that COVID-19 was created intentionally by humans, was measured by two scales, each consisting of three items, adopted from Imhoff and Lamberty (2020). The three items in the HOAX subscale are: “The virus is intentionally presented as dangerous in order to mislead the public,” “Experts intentionally mislead us for their benefit, even though the virus is not worse than a flu,” and “We should believe experts when they say that the virus is dangerous” (reverse-coded). The three items used in the CREATED subscale are: “Corona was intentionally brought into the world to reduce the population,” “Dark forces want to use the virus to rule the world,” and “I think it’s nonsense that the virus was created in a laboratory” (reverse-coded). The Czech translation of the original English scales was confirmed by a back translation.

### Participants and Data Collection

The convenience sample consisted of 866 students (mean age 23.58 years; 621 women) of medicine, law, and pedagogy at the universities located in Pilsen, Czech Republic. Out of the original 914 responses, seven participants were excluded as they did not belong to the studied population and 40 submissions were excluded as duplicates (for details, refer to Pisl et al., 2021). The participants were delivered a link to an online questionnaire presented *via* Google Forms from their lecturers, consisting of the above-described scales. To avoid any possible effects of priming or self-stylization with respect to the COVID-19-related beliefs that might possibly influence the responses to the DES and CMQ, the questions regarding COVID-19 were placed at the end of the questionnaire, and coronavirus was not mentioned in the introduction of the aims of the research. The study was approved by the Ethics Committee of the University Hospital and Faculty of Medicine in Pilsen (No. 49/2021), Czech.

### Settings

Data were collected between January 8 and January 21, 2021, during the second pandemic peak in the Czech Republic, shortly after vaccines were introduced and before they were made available to the general population. The first dose of the vaccine was given to a politician on December 27, 2020, and between then and January 21, 2021, 175,999 inhabitants, or 1.7% of the population received at least one dose (Mathieu et al., 2021). As of January 21, 2021, 15,445 persons died of coronavirus in the country of 10 million, according to government statistics (MZCR, 2021), with a mean of 164 daily deaths during the data collection period. According to a longitudinal panel survey with a sample representative of the Czech population above 15 years old, 78.4% of people of Czechs knew someone who was or had been ill with COVID-19 (National Pandemic Alarm, 2021). The pandemic-related concerns were rising since the previous November, together with the increasing perceived personal impact of the restrictions and dropping trust in the government (National Pandemic Alarm, 2021). The students were attending their lectures online and the national state of emergency was, except for 2 weeks before Christmas, in effect since October 2020, together with a night curfew, a general stay-at-home order, and the closure of many industries, such as hospitality, entertainment, and sport.

### Statistical Analysis

The scores for each scale were calculated as the sums of all items for scales of HLOC and CRT and as means for DES, CM, EHEALS, and (converting the reverse-scored questions) CC_HOAX, and CC_CREATED. In CRT, the inputs not containing any answer were interpreted as lack of effort rather than lack of ability to solve the puzzle and labeled as the missing values rather than the incorrect answers. A forced entry multiple linear regression analysis was used to evaluate the effect of the independent variables on the vaccination intentions in three different models, using the function “lm()” with its predefined parameters. For testing the interactions, moderation analysis was used as described by Wu and Zumbo (2008). The analysis was conducted in R 3.6.3, using the packages tidyverse (Wickham et al., 2019), psych (Revelle, 2020), and QuantPsyc (Fletcher, 2012); the figures were created using sjPlot (Lüdecke, 2021).

## Results

### Descriptive Statistics

In the sample of 866 university students, 65.70% reported the probability that they would get vaccinated against COVID-19 as higher than 50%; the mean reported probability was 67.48%. Further descriptive values are depicted in Table 1. As shown in Table 2, all the scales used had at least acceptable reliability, especially taking into consideration the low number of items of some scales, and intercorrelations found elsewhere (refer to, for instance, Wallston, 2004).

**TABLE 1:** Descriptive statistics.

	*n*	Min	Max	Mean	Med	Standard deviation	Standard Error	Skew	Kurtosis
DES	866	0	78.21	17.58	14.11	13.1	0.45	1.24	1.53
CM	866	2	100	56.04	58	20.21	0.69	–0.15	–0.47
HOAX	866	0	100	23.60	16.67	24.22	0.82	0.95	0
CREATED	866	0	100	29.77	26.67	21.76	0.74	0.66	–0.11
EHEALS	866	1	5	3.85	4	0.82	0.03	–0.66	0.07
CRT	842	0	3	1.51	2	1.19	0.04	–0.05	–1.52
HLOC_I	866	11	36	25.04	25	4.27	0.15	–0.24	0.15
HLOC_C	866	6	34	16.19	16	4.93	0.17	0.4	0.18
HLOC_P	866	6	34	19.60	20	4.65	0.16	–0.15	–0.09
VAC	866	0	100	67.48	80	33.63	1.14	–0.73	–0.89

*DES, Dissociation Experience Scale; CM, conspiracy mentality; HOAX, a conspiracy theory that COVID-19 is a hoax; CREATED, a conspiracy theory that COVID-19 is human-made; EHEALS, digital health literacy; CRT, cognitive reflection test; HLOC_I, HLOC_C, and HLOC_P, dimensions of health locus of control: internal, chance, and powerful others; VAC, willingness to get vaccinated.*

**TABLE 2:** Correlation matrix.

	Cronbach’s alpha (Number of items)	DES	CM	HOAX	CREATED	EHEALS	CRT	HLOC_I	HLOC_C	HLOC_P
DES	0.93 (28)									
CM	0.82 (5)	0.33								
HOAX	0.88 (3)	0.15	0.30							
CREATED	0.67 (3)	0.17	0.42	0.46						
EHEALS	0.92 (8)	–0.08	–0.06	–0.14	–0.11					
CRT	0.73 (3)	–0.16	–0.19	–0.21	–0.25	0.05				
HLOC_I	0.67 (6)	0.02	0.05	0.05	–0.02	0.23	0.02			
HLOC_C	0.69 (6)	0.17	0.09	0.10	0.13	–0.06	–0.09	–0.10		
HLOC_P	0.66 (6)	0.02	–0.04	–0.26	–0.14	0.04	0.06	0.08	0.23	
VAC	NA (1)	–0.09	–0.25	–0.60	–0.41	0.12	0.20	–0.04	–0.04	0.30

*DES, Dissociation Experience Scale; CM, conspiracy mentality; HOAX, a conspiracy theory that COVID-19 is a hoax; CREATED, a conspiracy theory that COVID-19 is human-made; EHEALS, digital health literacy; CRT, cognitive reflection test; HLOC_I, HLOC_C, and HLOC_P, dimensions of health locus of control: internal, chance, and powerful others; VAC, willingness to get vaccinated.*

### Model 1

**FIGURE 1:**
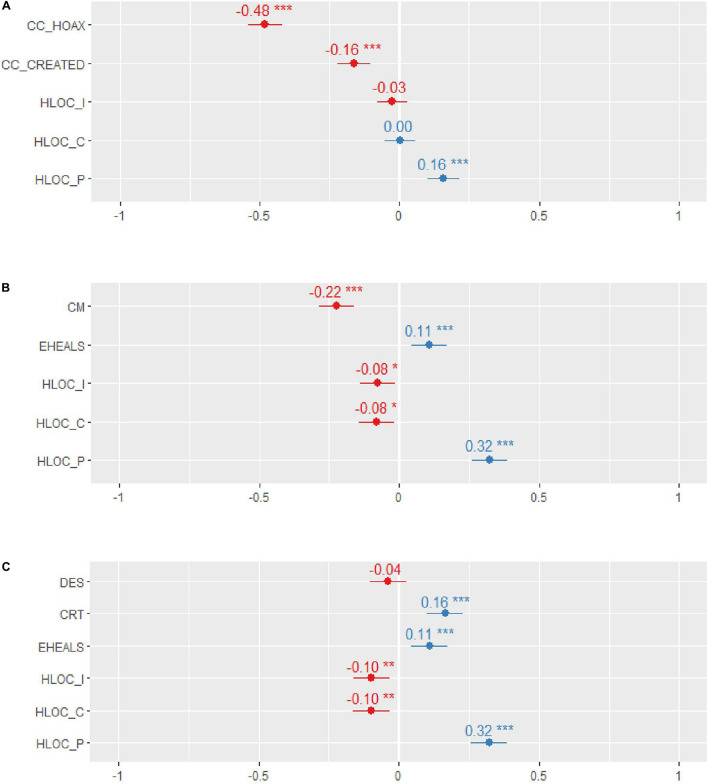
The effects of predictors on the willingness to get vaccinated. Beta coefficients of variables predicting willingness to get vaccinated in the linear regression model 1 **(A)**, 2 **(B)**, and 3 **(C)**. CC_HOAX, a conspiracy theory that COVID-19 is a hoax; CC_CREATED, a conspiracy theory that COVID-19 is human-made; CM, conspiracy mentality; EHEALS, digital health literacy; DES, Dissociation Experience Scale; CRT, cognitive reflection test; HLOC_I, HLOC_C, and HLOC_P, internal, chance, and powerful others dimension of health locus of control. *p < 0.05, **p < 0.01, and ***p < 0.001.

A multiple regression model using belief in COVID-19-related conspiracies and three dimensions of HLOC as predictors explained 40.21% of the variance of willingness to get vaccinated [*R*^2^ = 0.40, *F*(5,860) = 115.70, *p* < 0.001]. VAC was most strongly predicted by the belief that COVID-19 is a hoax (β = − 0.67, *p* < 0.001), followed by the belief that COVID-19 was created (β = − 0.16, *p* < 0.001), and *powerful others* HLOC (β = 0.16, *p* < 0.001), while the other two dimensions of HLOC had no effect (both the values of *p* > 0.3). The results summarized in Table 3 and visualized in Figure 1A support our hypothesis about VAC being predicted by the belief in the COVID-19-related conspiracy theories and reveal that when conspiracy theories about COVID-19 are taken into account, VAC is predicted by *powerful others*, but not internal or chance HLOC.

**TABLE 3:** The multivariate linear regression predicting the vaccination intentions (Models 1–3).

	MODEL 1				MODEL 2				MODEL 3			
*Predictors*	*Estimates*	*Beta*	*t*	*p*	*Estimates*	*Beta*	*t*	*p*	*Estimates*	*Beta*	*t*	*p*
(Intercept)	73.15	–0.00	10.47	**< 0.001**	50.24	–0.00	5.46	**< 0.001**	29.81	0.00	3.26	**0.001**
CC_HOAX	–0.67	–0.48	–15.52	**< 0.001**								
CC_CREATED	–0.25	–0.16	–5.45	**< 0.001**								
HLOC_I	–0.20	–0.03	–0.97	0.332	–0.61	–0.08	–2.41	**0.016**	–0.77	–0.10	–2.97	**0.003**
HLOC_C	0.02	0.00	0.10	0.919	–0.56	–0.08	–2.54	**0.011**	–0.66	–0.10	–2.91	**0.004**
HLOC_P	1.14	0.16	5.51	**< 0.001**	2.33	0.32	10.02	**< 0.001**	2.31	0.32	9.74	**< 0.001**
CM					–0.37	–0.22	–7.14	**< 0.001**				
EHEALS					4.38	0.11	3.36	**0.001**	4.51	0.11	3.36	**0.001**
DES									–0.10	–0.04	–1.19	0.236
CRT									4.62	0.16	5.07	**< 0.001**
Observations	866				866				842			
*R*^2^/*R*^2^ adjusted	0.402/0.399				0.172/0.168				0.155/0.149			

*Estimates, beta, unstandardized and standardized regression coefficient; HOAX, a conspiracy theory that COVID-19 is a hoax; CREATED, a conspiracy theory that COVID-19 is human-made; HLOC_I, HLOC_C, and HLOC_P, dimensions of health locus of control: internal, chance, and powerful others; CM, conspiracy mentality; EHEALS, digital health literacy; DES, Dissociation Experience Scale; CRT, cognitive reflection test; VAC, willingness to get vaccinated; *p*-values < 0.05 in bold.*

### Model 2

A multiple regression model using CM, digital health literacy, and three dimensions of HLOC as predictors explained 17.23% of the variance of willingness to get vaccinated [*R*^2^ = 0.17, *F*(5,860) = 35.81, *p* < 0.001]. VAC was most strongly predicted by *powerful others* HLOC (β = 0.32, *p* < 0.001), followed by CM (β = − 0.22, *p* < 0.001), digital health literacy (β = 0.11, *p* < 0.001), *internal* (β = − 0.08, *p* < 0.05), and *chance* (β = − 0.08, *p* < 0.05) HLOC. The results summarized in Table 3 and visualized in Figure 1B support our hypotheses about VAC being related to the CM and digital health literacy. Further, the results reveal that when CM and digital health literacy are taken into account, VAC is linked to low *internal* HLOC (contrary to our expectations), high *powerful others* HLOC, and low *chance* HLOC.

The moderation analysis was used to test whether the HLOC_I effect on the vaccination intentions may be moderated by the CM or digital health literacy (EHEALS). To test this, the HLOC_I and VAC scores were centered and scaled, and a regression model predicting CM (or EHEALS, respectively) based on the HLOC_I and VAC was compared with the same model containing the product of HLOC_I and VAC. The results of ANOVA revealed that the models did not differ significantly, indicating that there was no significant moderation effect of either EHEALS [*F*(1,862) = 1.85, *p* = 0.17] or CM [*F*(1,862) = 1.63, *p* = 0.20] on the link between HLOC_I on VAC. Therefore, our hypothesis that the effect of HLOC_I on vaccination intentions (VAC) may be moderated by CM and/or EHEALS is not supported by the data.

### Model 3

A multiple regression model using experience with dissociation, cognitive reflection, digital health literacy, and three dimensions of HLOC as predictors explained 15.53% of the variance of the willingness to get vaccinated [*R*^2^ = 0.16, *F*(5,860) = 25.58, *p* < 0.001]. VAC was most strongly predicted by *powerful others* HLOC (β = 0.32, *p* < 0.001), followed by cognitive reflection (β = 0.22, *p* < 0.001), digital health literacy (β = 0.11, *p* < 0.001), *internal* (β = − 0.10, *p* < 0.01), and *chance* (β = − 0.10, *p* < 0.01) HLOC, while the effect of dissociation was not significant (*p* = 0.23). The results summarized in Table 3 and visualized in Figure 1C confirm the hypothesized effect of cognitive reflection on VAC but not the effect of dissociation on VAC. Further, they reveal that when dissociation and cognitive reflection are taken into account, VAC is linked to high *powerful others* HLOC, low *chance* HLOC, and low *internal* HLOC.

The results indicate that a one SD increase of *powerful others* dimension of HLOC was linked to an additional 10.76% of the subjectively estimated probability that the individual was going to get vaccinated against COVID-19. Similarly, a one SD increase in cognitive reflection and digital health literacy was linked to an additional 5.38% (CRT) and 3.70% (EHEALS) of the subjectively estimated probability that the individual was going to get vaccinated, and a decrease of one SD in *internal* or *chance* dimensions of HLOC was linked to an additional 3.36% of the subjectively estimated probability of getting vaccinated.

## Discussion

The presented data support the hypotheses that the willingness to get vaccinated against COVID-19 is reduced by the belief in COVID-19-related conspiracy theories and its predictors: CM, low digital health literacy, and low cognitive reflection. Experience with dissociation had no effect on the willingness to get vaccinated. The vaccination intentions were strongly positively related to the *powerful others* dimension of HLOC and negatively to *chance* HLOC. Contrary to our expectations, *internal* HLOC also reduced the vaccination intentions and the effect of *internal* HLOC was not moderated by CM or digital health literacy.

The result showed that 66% of Czech university students participating in our study were willing to get vaccinated. Our result is consistent with the previous findings that 60–79% of the population was going to get vaccinated worldwide in the summer of 2020 and that the willingness to get vaccinated against COVID-19 was decreasing through the year 2020, with Eastern Europe (represented by Poland) showing the lowest vaccination intentions (Robinson et al., 2021). Considering the national representative survey with 50.3–50.4% of Czechs planning vaccination against COVID-19 (National Pandemic Alarm, 2021), our university student sample was showing above-average vaccination intentions, suggesting that the positive effect of higher education on the willingness to get vaccinated (Schwarzinger et al., 2021) was stronger than the negative effect of younger age (Neumann-Böhme et al., 2020; Robinson et al., 2021). It may also support the findings of some studies that the relationship between the vaccination intentions and age may be “U”-shaped rather than linear, with the middle-aged being least willing to get vaccinated (Kourlaba et al., 2021; Schwarzinger et al., 2021).

The vaccination intentions were predicted by COVID-19-related conspiracy theories. Further, they were predicted by digital health literacy, CM, and cognitive reflection. This is consistent with the previous research revealing a positive link between the vaccination intentions and cognitive reflection as a proxy of analytical cognitive style (Murphy et al., 2021). The observed effect of cognitive reflection on the vaccination intentions also mimics the results of an experimental study showing that promoting rational decision-making increases the intentions to wear a face mask (Capraro and Barcelo, 2021), indicating that our observations may be used to inspire interventions. Health literacy was also found to be predictive of higher vaccination intake under the conditions of a high risk of getting sick and complications in the short-term (Lorini et al., 2018)—conditions which are certainly satisfied with respect to the current pandemic. The experience with dissociation was not predictive of vaccination intention, even though it was predictive of CM (Pisl et al., 2021) which, in turn, predicted lower vaccination intentions. Given that the effect of paranormal thinking on belief in conspiracy theories is reduced by education (Douglas et al., 2016), the expected negative effect of dissociation experience on the vaccination intentions might possibly be present in the general population, although it was not reflected in our highly educated sample of university students. Alternatively, it is possible that while dissociation increases belief in the conspiracy theories, its effect does not translate into the changes in attitude toward vaccination.

The *powerful others* dimension of HLOC was strongly positively related to the intention of getting vaccinated, while the two other dimensions of HLOC (*internal* and *chance*) were related to vaccination weakly and negatively. A recent study found the same pattern of the effects of HLOC on the vaccine intentions in British, but not in an Irish representative sample of the general adult population (Murphy et al., 2021). Our data are also consistent with the previous findings that *powerful others* HLOC is positively related to pro-vaccination attitudes in parents (Tinsley and Holtgrave, 1989; Aharon et al., 2018) and nurses (Zhang et al., 2012; Kan et al., 2018), even though no effect of HLOC was found with respect to influenza vaccination in the elderly (Nexøe et al., 1999). Given that *powerful others* HLOC correlates with trust in the physicians (Brincks et al., 2010) and concerns related to side-effects and safety of vaccines are the top reasons for vaccine hesitation and refusal (Neumann-Böhme et al., 2020), the link between HLOC and willingness to get vaccinated may be mediated by trust in the medical professionals.

The *chance* HLOC was negatively related to the intention to get vaccinated in the latter two models, which is again consistent with the attitudes of parents toward vaccination (Aharon et al., 2018) as well as recent findings that the *chance* HLOC partly mediates the negative relationship between the religiosity and vaccination intentions (Olagoke et al., 2021). The absence of effect of *chance* HLOC in the first model, when two particular conspiracy theories were included, might reflect the correlation between the conspiracy beliefs and external HLOC in general (Abalakina-Paap et al., 1999).

The *internal* dimension of HLOC predicted the lower vaccination intentions in the latter two models. This is contrary to the model based on the attitudes of parents toward vaccination (Aharon et al., 2018) but consistent with the recent findings from Great Britain and Ireland (Murphy et al., 2021). The patients with higher *internal* HLOC might be more prone to follow their judgment rather than the advice of the professional community, as vaccine hesitancy may be an act of self-empowerment (Velan, 2016). In such cases, we would expect the link between the *internal* HLOC and vaccination intentions to be moderated by digital health literacy and/or CM, as it would be those individuals who lack health literacy and/or are prone to conspiracy thinking, for whom high *internal* HLOC would result in vaccine hesitancy. However, such moderation was not found in our data. Noticing that the findings of negative associations between *internal* HLOC and the willingness to get vaccinated come from highly informed samples, considering the medialization of COVID-19 in our study and in Murphy et al. (2021) and the medical background of the sample of Kan et al. (2018), we propose that *internal* HLOC may increase the vaccination intentions in the less-informed populations (perhaps increasing their awareness of the benefits or the mere existence of the vaccine) and decrease it in more informed ones (perhaps increasing the safety or efficacy concerns).

Our results may serve as a warning that promoting *internal* HLOC with respect to COVID-19 might come with an adverse effect on the willingness to get vaccinated. This is relevant, because the *internal* HLOC was previously found to be related to higher information seeking and lower depression, anxiety, and stress symptoms during the pandemic, and promoting it was suggested to reduce the psychiatric burden of COVID-19 (Sigurvinsdottir et al., 2020). This might be especially relevant for younger populations, as *internal* HLOC tends to decrease with age, together with a decreasing capacity to influence the health outcomes of an individual (Bailis et al., 2010). With respect to this age-specific pattern, the negative effect of *internal* HLOC on the willingness to get vaccinated might reflect overestimating the ability of an individual to cope with COVID-19 or reducing complacency in terms of the Confidence, Complacency, and Convenience Model of Vaccine Hesitancy (WHO, 2014). Such explanations would fit the finding that considering COVID-19 harmless is the third most popular reason for refusing vaccination in Europe (after concerns about vaccine side-effects and safety; Neumann-Böhme et al., 2020).

Altogether, 40% of the variance of vaccination intentions are explained by the belief in the COVID-19-related conspiracy theories and *powerful others* HLOC. Our final model then explained 16% of the variance of vaccination intentions based on cognitive reflection, digital health literacy, and HLOC. HLOC (the *powerful others* dimension in particular) was found to have the largest effect on the vaccination intentions, followed by the cognitive reflection, EHEALS, and the other two dimensions of HLOC. The effect of dissociation experience was not confirmed.

### Recommendations

Reducing proneness to believing in the conspiracy theories by increasing analytical thinking and digital health literacy may increase the willingness to comply with the recommendations to get vaccinated in general. In the short-term, disproving the COVID-19-related conspiracy theories may have a positive effect on the willingness to get vaccinated against COVID-19. Furthermore, the positive link between *powerful others* HLOC and the willingness to get vaccinated suggests that the campaigns promoting vaccinations should target especially those not connecting their health with other persons. Because the persuasiveness of health-related promotion campaigns is increased when matching the prevailing HLOC of an audience (Williams-Piehota et al., 2004), the promotional messages should be created to appeal to audiences deriving their health from internal decisions (“Vaccination – your gift to yourself!”) or chance and destiny (“Destined to get vaccinated!”), rather than to those connecting their health with *powerful others* (“Scientists and doctors say: get vaccinated!”). Furthermore, the attempts to promote vaccination against COVID-19 should target those with intuitive rather than analytical cognitive style has given the lower vaccination intentions in those with low cognitive reflection.

### Further Research

Possible mediators of the effect of *powerful others* HLOC on the vaccination intentions should be examined. To find ways to increase the intentions to get vaccinated, it would be beneficial to learn whether the effect of HLOC, which is relatively stable and developed in childhood (Lau, 1982), on the vaccination intentions may be mediated by something readier to change, such as the trust in health professionals. The effect of *internal* HLOC on the vaccination intentions and other health attitudes remains elusive and should be studied, especially with relation to how well subjects are informed about the scrutinized subject.

### Limitations

The timing of the data collection in the weeks after the vaccination campaign was started limits the generalization of the absolute numbers. The immediate effect of pandemics on individual lives may strengthen the political and epistemic predictors of the conspiratorial explanations at the expense of the psychological ones (Hartman et al., 2020), which may have affected the attitudes toward vaccination. Further, the weekly number of persons met for at least 5 min in person was reduced to 17–18 in the respective period according to data on a representative Czech sample between 18 and 34 years of age (compared with up to 29.5 when the restrictions were loosened in summer 2020; Zivot behem pandemie, 2021). This might have affected our estimate of the relative importance of personal predictors and social factors with respect to the vaccination intentions. For instance, the personal willingness to get vaccinated is positively associated with the estimated vaccination intentions of peers and society (Agranov et al., 2021; Graupensperger et al., 2021). With peer interactions taking place online and offline (Luo et al., 2021), the effects of restricting personal contacts on social factors are complex, limiting the generalization of our findings beyond the end of the pandemic restrictions. Besides the restrictions, social processes tend to be affected by the experience of a disastrous event such as a pandemic (Sullivan, 2014; Townshend et al., 2015), which might have, again, affected the conspiracy beliefs as well as the willingness to comply with the recommendations to get vaccinated in a manner specific for a given time and place. Further, the survey answers of the participants about willingness to vaccinate might differ from their actual decision. For example, in a study of Dutch healthcare professionals, only 73.9% of those reporting high intention to get vaccinated against influenza in a survey were vaccinated a month later (compared with 1.3% of those with no intention; Lehmann et al., 2014).

The sample of university students may have influenced the effects of the scrutinized factors on the beliefs in conspiracy theories, *via* the above-mentioned effects of age and education on the willingness to get vaccinated and by the heterogeneity related to different fields of their studies. Also, our sample included predominantly (72%) female participants and women who have lower vaccination intentions and acceptance than men across the countries (Wang et al., 2021; Zintel et al., 2021), with the effect of gender being partly mediated by perceived behavioral control in the British and German samples (Sieverding et al., 2021). Because perceived behavioral control is conceptually related to HLOC, this might have affected the observed effects, although the link between both the constructs is weak, with HLOC explaining only 4% of the variance in the perceived behavioral control (Armitage, 2003). Only two COVID-19-related conspiracy theories were used for the analysis, limiting its generalizability to the whole scope of conspiracy beliefs about coronavirus.

## Conclusion

In the study, two-thirds of our sample of Czech university students were willing to get vaccinated in January 2021, outpacing the national average of 50% of the population. About 40% of the variance of the willingness to get vaccinated was explained by *powerful others* HLOC and two conspiracy beliefs, indicating that a substantial part of vaccine refusal is a consequence of individual beliefs and characteristics rather than a moral decision one can be blamed for. One-sixth of the variance of vaccination intentions was explained by cognitive reflection, digital health literacy, and—especially—HLOC, showing that the psychological variables are relevant for the willingness to get vaccinated against COVID-19. The understanding of the predictors of vaccination intentions should be reflected in the campaigns promoting vaccination against COVID-19.

## Data Availability Statement

The raw data supporting the conclusions of this article will be made available by the authors, without undue reservation.

## Ethics Statement

The studies involving human participants were reviewed and approved by Ethics Committee of the University Hospital and the Faculty of Medicine, Charles University in Pilsen (49/2021, 4th February 2021). The patients/participants provided their written informed consent to participate in this study.

## Author Contributions

JVe, JVo, and VP: conceptualization. VP, JVe, and EC: methodology. VP and EC: formal analysis. VP, JVe, and GK: investigation. JVe, GK, KC, and VP: resources. VP: writing—original draft preparation. JVo, JVe, EC, GK, KC, and VP: writing—review and editing. JVe: supervision, project administration, and funding acquisition. All authors contributed to the article and approved the submitted version.

## Conflict of Interest

The authors declare that the research was conducted in the absence of any commercial or financial relationships that could be construed as a potential conflict of interest.

## Publisher’s Note

All claims expressed in this article are solely those of the authors and do not necessarily represent those of their affiliated organizations, or those of the publisher, the editors and the reviewers. Any product that may be evaluated in this article, or claim that may be made by its manufacturer, is not guaranteed or endorsed by the publisher.
